# Executive dysfunction and cortical variations among intimate partner violence perpetrators and the association with sexism

**DOI:** 10.1093/scan/nsae046

**Published:** 2024-06-25

**Authors:** Ángel Romero-Martínez, María Beser-Robles, Leonor Cerdá-Alberich, Fernando Aparici, Luis Martí-Bonmatí, Carolina Sarrate-Costa, Marisol Lila, Luis Moya-Albiol

**Affiliations:** Department of Psychobiology, University of Valencia, Valencia 46010, Spain; Biomedical Imaging Research Group (GIBI230), La Fe Health Research Institute, Valencia 46026, Spain; Biomedical Imaging Research Group (GIBI230), La Fe Health Research Institute, Valencia 46026, Spain; Biomedical Imaging Research Group (GIBI230), La Fe Health Research Institute, Valencia 46026, Spain; Biomedical Imaging Research Group (GIBI230), La Fe Health Research Institute, Valencia 46026, Spain; Department of Psychobiology, University of Valencia, Valencia 46010, Spain; Department of Social Psychology, University of Valencia, Valencia 46010, Spain; Department of Psychobiology, University of Valencia, Valencia 46010, Spain

**Keywords:** cortical thickness, intimate partner violence perpetrators, neuroimaging, magnetic resonance, prefrontal cortex, sexism

## Abstract

Malfunctioning in executive functioning has been proposed as a risk factor for intimate partner violence (IPV). This is not only due to its effects on behavioral regulation but also because of its association with other variables such as sexism. Executive dysfunctions have been associated with frontal and prefrontal cortical thickness. Therefore, our first aim was to assess differences in cortical thickness in frontal and prefrontal regions, as well as levels of sexism, between two groups of IPV perpetrators (with and without executive dysfunctions) and a control group of non-violent men. Second, we analyzed whether the cortical thickness in the frontal and prefrontal regions would explain sexism scores. Our results indicate that IPV perpetrators classified as dysexecutive exhibited a lower cortical thickness in the right rostral anterior cingulate superior frontal bilaterally, caudal middle frontal bilaterally, right medial orbitofrontal, right paracentral, and precentral bilaterally when compared with controls. Furthermore, they exhibited higher levels of sexism than the rest of the groups. Most importantly, in the brain structures that distinguished between groups, lower thickness was associated with higher sexism scores. This research emphasizes the need to incorporate neuroimaging techniques to develop accurate IPV profiles or subtypes based on neuropsychological functioning.

## Introduction

During the last years, there has been a growing recognition of the importance for clinicians and scholars to join efforts to drastically reduce the number of women who are victims of violence perpetrated by their male partners, known as intimate partner violence (IPV) (Sardinha et al. 2022, Lila and Gilchrist 2023). Some of these professionals have attempted to assess the risk profiles of IPV perpetrators based on certain risk variables (e.g. personality disorders, drug misuse or sexism) associated with men convicted of IPV. Theoretically, developing risk profiles for these men can help guide interventions by including modules that focus on the therapeutic needs of IPV perpetrators. This, in turn, could help reduce the premature abandonment of interventions (dropout). Additionally, by increasing IPV perpetrators adherence to treatment (or its completion) the risk of recidivism tends to be reduced (Santirso et al. 2020). However, most of the research these findings are based on primarily used self-reports, qualitative instruments and, in some cases, neuropsychological tests (Pinto et al. 2010, Horne et al. 2020, Humenik et al. 2020, Stover et al. 2022). Despite the value and rigor of this research, the inherent limitations of these tools highlight the need to introduce new instruments, such as neuroimaging techniques (e.g. analyzing the cortical thickness), to overcome the limitations of psychological instruments and to join efforts to gain a more accurate understanding of the complex phenomenon of IPV.

Studies of injuries in the frontal lobe (the famous case of Phineas Gage, sequelae seen in combat veterans, people with intermittent explosive disorder, traumatic brain injuries, etc.) or naturally occurring variations or individual differences in cortical thickness, concretely, in prefrontal structures, indicate an increased likelihood of these individuals engaging in violent, impulsive, and criminal acts (Chester et al. 2017, Lamsma et al. 2017, Padrón et al. 2022, Sheehan et al. 2021, Yang and Raine 2009, Zhu et al. 2020). In this regard, an inverse association between the cortical thickness of prefrontal regions and proneness to aggression has been established (Bounoua et al. 2020). However, despite the significant role played by prefrontal cortex variations in cortical thickness when it comes to violence proneness, the exact mechanisms that explain this tendency remain unknown. For example, from a neurological perspective, it has been hypothesized that prefrontal structures serve as a system for behavioral control. Hence, damages or dysfunctions in these brain structures can result in an inability to regulate limbic dysregulation (e.g. exaggerated response to facial stimulus, alterations in emotion regulation, and/or expression, dysfunctions in motivational processes, among others) which, under certain circumstances, can contribute to behavioral instability and violence. That is, violence mainly driven by emotions and frequently unplanned (Romero-Martínez et al. 2019, 2020, Nikolic et al. 2022). Nonetheless, it would be appropriate to enhance this model by adding conclusions derived from a neuropsychological perspective. This will provide a comprehensive understanding of how prefrontal variations in cortical thickness can contribute to violence through their role in cognitive processes.

Neuropsychological scientists have suggested that prefrontal variations (e.g. morphological and/or functional) contribute to violence. This is because these brain structures are involved in a set of cognitive processes known as executive functioning. The greater the prefrontal cortex volume the better the executive functioning (Yuan and Raz 2014). These cognitive domains comprise a broad range of cognitive abilities, such as cognitive flexibility, planning abilities, working memory, among others. All of these abilities are necessary to properly maintain cognitive control and adjust behavior to demanding contexts (Diamond 2013). Consequently, individuals with executive dysfunctions have serious difficulties when trying to solve problems by switching their strategies to other more appropriate ones or even maintaining certain cognitive schemas. They exhibit a rigid behavior, with serious difficulties to regulate or anticipate consequences of impulsive outbursts (Cruz et al. 2020). Therefore, it makes sense to conclude that individuals with executive dysfunctions (e.g. low cognitive flexibility and planning abilities, working memory deficits, among others) exhibit a heightened risk of engaging in violent acts (Cruz et al. 2020).

Regarding IPV perpetrators, research in this field allows us to conclude that executive dysfunctions are involved in IPV perpetration, although they are not necessarily the main factors or direct factors that explain it (Vitoria-Estruch et al. 2017, 2018, Horne et al. 2020, Humenik et al. 2020). In terms of executive functioning processes, much of the research has concluded that reduced cognitive flexibility allows to distinguish IPV perpetrators from non-violent men, with men convicted of IPV exhibiting a slightly lower functioning than control groups. Furthermore, a reduced cognitive flexibility, along with other executive functioning alterations, has also been associated with recidivism and the persistence of sexist schemas (Vitoria-Estruch et al. 2017, 2018, Romero-Martínez et al. 2021, 2022b). However, the above-mentioned empirical research did not link cognitive alterations to cortical thickness in the frontal structures of IPV perpetrators.

If we take a look at cortical thickness in terms of frontal and prefrontal structures, a previous study concluded that IPV perpetrators exhibited a smaller cortical thickness in the orbitofrontal cortex (bilaterally) and the right anterior cingulate cortex when compared to men convicted of other types of crimes (Verdejo-Román et al. 2019). Unfortunately, they did not include a control group (non-convicted and/or non-violent men) and did not indicate whether the differences in cortical thickness in this brain region were responsible for executive dysfunctions. Hence, it is necessary to conduct additional empirical research to establish a clear link between executive functioning and variations in cortical thickness in frontal and prefrontal brain structures. Establishing an association between these brain structures might help to corroborate the extension and meaning of executive functioning in IPV perpetrators, which is mainly based on their performance in neuropsychological tests.

As far as we know, only a limited number of studies have measured brain variations (e.g. volume differences, and functional connectivity) in IPV perpetrators, as violent and convicted individuals, compared to other groups of men without this type of conviction (Bueso-Izquierdo et al. 2022). Nevertheless, only one single study considered whether the cortical thickness of the frontal and prefrontal structures allowed to distinguish IPV perpetrators with and without executive dysfunctions and control men. Thus, the main objective of this study was to analyze differences in cortical thickness in frontal and prefrontal structures between two groups of IPV perpetrators (with and without executive dysfunction) and a group of non-violent individuals. We first hypothesized that, in line with previous research (Vitoria-Estruch et al. 2017, 2018, Verdejo-Román et al. 2019, Horne et al. 2020, Humenik et al. 2020, Romero-Martínez et al. 2021, 2022b), IPV perpetrators who presented executive dysfunctions would exhibit lower cortical thickness in the prefrontal cortex (e.g. dorsolateral, ventrolateral and orbitofrontal) and anterior cingulate cortex, along with higher sexism rates than IPV perpetrators without executive dysfunctions. In addition, that cortical thickness would be markedly different from the control group. The second objective of this study was to evaluate whether variations in the cortical thickness of the frontal and prefrontal regions are related to higher scores in sexism (e.g. persistent cognitive schemas). As previously stated in the scientific literature, a smaller thickness in certain frontal areas can lead to low mental flexibility and the maintenance of rigid cognitive schemas when solving problems (Burzynska et al. 2012, Kollndorfer et al. 2023, Raine et al. 2012), which tends to be associated with sexism (Vitoria-Estruch et al. 2017, 2018). Therefore, we expect that a reduced thickness of the frontal, specifically prefrontal, regions would be associated with higher sexism scores, when controlling for executive functioning.

## Method

### Participants

After screening a total of 120 participants who voluntarily accepted to participate in the study by signing the informed consent, a total of 117 participants (60 IPV perpetrators and 57 controls) were finally included, given that three participants of the control group showed a strong indication of a dysexecutive syndrome.

Regarding IPV perpetrators, the sample of men was gathered from the psychological and psychoeducational community treatment program called “CONTEXTO”, which is developed and implemented by our university. This intervention is mandatory for men who receive a sentence of <2 years in prison for gender violence against their intimate partner and who have no previous criminal records. In particular, these men are offered this intervention as an alternative to completing their sentence. The intervention program is mandatory and must be completed (Lila et al. 2018). In addition, men who agreed to participate, were interviewed by two independent researchers before starting the intervention program to verify the absence of physical (e.g. brain damage, chronic pain, mild and/or severe cranioencephalic trauma with a temporary loss of consciousness—minutes to days) or mental (mood, personality, psychotic disorders, drug use disorder, etc.) disorders and an intelligence quotient (IQ) ≥80, as well as the absence of claustrophobia and Class III obesity (for further details see Romero-Martínez. et al. 2024). The participation rate of IPV perpetrators from each therapeutic group of IPV perpetrators ranged from 20% to 30%.

With regards to the control group, 60 men who had no previous criminal records (including IPV or any kind of violence) were finally included in our research study. The recruitment process of these participants was based on advertisements published in the city of Valencia (Spain), as well as on several social media platforms. All participants who showed interest were initially screened during a telephone interview. Afterwards, they were given an appointment at the University for two independent researchers to check that they met the inclusion criteria. These criteria were to have no criminal record of violence against their partner or another individual, which was verified based on a criminal record certificate issued by a public institution and voluntarily provided by participants. Furthermore, it was necessary to score below 1 on the psychological aggression, physical assault, and sexual coercion subscales of the Conflict Tactics Scale-II (Straus et al. 1996, Muñoz-Rivas et al. 2007). Additionally, they could not suffer from any physical or mental disorder and had to have an IQ ≥80 (Kaufman and Kaufman 1997).

All participants were classified into the dysexecutive (those with executive dysfunctions) or non-dysexecutive group according to their performance on different neuropsychological tests assessing executive functioning, such as the Wisconsin Card Sorting Test (number of perseverative errors) (Heaton et al. 1993), part B of the Hayling test (Burgess and Shallice 1997), F–A–S Test phonemic and semantic fluency (Del Ser Quijano et al. 2004) and the Key Search Test of the Behavioural Assessment of the Dysexecutive Syndrome (BADS) (total score) (Wilson et al. 1996). To be classified as dysexecutive, it was necessary to have a percentile below 50 in at least three out of the five scales employed. This was considered a strong indication of dysexecutive syndrome. Accordingly, IPV perpetrators were divided into dysexecutive (*n* = 38) and non-dysexecutive (*n* = 22). Three participants of the control group showed a strong indication of dysexecutive symptoms. These participants were excluded from the statistical analysis, leaving a total of 57 participants in the control group.

The experiment was carried out following the ethical and legal guidelines of the Helsinki Declaration and was approved by the University Ethics Committee (code: H1515749368278).

### Procedure

Men who showed interest in participating in our study were initially informed of the main objectives and requirements of the study during a phone call. During this call, they were screened using several questions (e.g. age, body mass index, claustrophobia, cranioencephalic metallic implants, among others). After ensuring their suitability for the study they were given an appointment for the first session in the psychobiology laboratories of the Faculty of Psychology and then a second session at La Fe Health Research Institute.

Those who agreed to participate signed an informed consent form and were also given an appointment for the session in the Psychology laboratories between 10 a.m. and 2 p.m. During this session, a semi-structured individual interview was conducted with two independent researchers who have vast experience treating and assessing IPV perpetrators to exclude those who did not meet the inclusion criteria, as well as to collect relevant information for the study (sociodemographic data, IQ, executive functioning, psychological variables, etc.). An agreement between both interviewers ≥0.70 (Cohen’s kappa) in each domain assessed was necessary.

After ending the session in the Psychology laboratories, participants were called back within 1 week to undergo a magnetic resonance imaging (MRI) at the University and Polytechnic Hospital of La Fe, which lasted ∼30 min. At the end of this phase, the individual was thanked for their participation and received €100 to cover dietary and travel expenses.

### Instruments

#### Neuropsychological instruments

The IQ calculation was based on the Spanish-validated version of the Kaufman Brief Intelligence Test (Kaufman and Kaufman 1997). This test is divided into two parts: one focuses on vocabulary (i.e. expressive vocabulary and definitions) and the other on matrices, which consist of a series of abstract figure sequences.

*Wisconsin Card Sorting Test (WCST)* was used to measure cognitive flexibility or the ability to effectively adjust to environmental challenges or demands. This test consists of four stimulus cards and 128 response cards containing various stimuli, such as colors, shapes, and numbers of figures (Heaton et al. 1993). For this study, we employed the number of trials, total errors, perseverative errors, and number of categories completed. Low cognitive flexibility or worse performance is characterized by a high number of trials and errors, as well as a low number of completed categories.

*For the assessment of verbal inhibition*, we employed the Hayling test (Burgess and Shallice 1997). This test, divided in two parts (section A and B), measures initiation speed and response suppression. In section A, participants need to quickly provide a verbal response to complete a sentence that is missing the last word, ensuring that the meaning is consistent. In section B, participants also need to complete the sentences but using an inconsistent word. Specifically, we employed the total score (or the number of correct responses) in part B as a dependent variable for this study. A higher total score is interpreted as higher verbal inhibition ability.

*F–A–S Verbal Phonemic Fluency Task* consists of two parts. In the first task, participants need to verbalize as many words as possible for each letter (F, A, and S). They have 60 s for each letter. This task assesses phonemic fluency or the ability to retrieve words with a specific phoneme. For verbal semantic fluency or the ability to retrieve words belonging to a specific category (e.g. animals), participants are asked to name as many animals as possible in 60 s. In both cases, the higher the total score in each part or number of words named, the better the verbal phonemic and semantic fluency (Del Ser Quijano et al. 2004).

*Key Search Test* is a subtest of the BADS (Wilson et al. 1996) and was used to measure planning abilities. Participants have to draw an itinerary to discover a lost key. For this test, the total score was employed, with a higher score indicating better planning abilities.

#### Psychological instruments

To measure the levels of IPV, a widely employed tool in this field of research was used; the Revised Conflict Tactics Scale (CTS2) (Straus et al. 1996) adapted to Spanish (Muñoz-Rivas et al. 2007). This test contains 78 items, which range from 0 (This has never happened) to 6 (More than 20 times in the past year). This study used the psychological aggression (e.g. I accused my partner of being a lousy lover, I shouted or yelled at my partner), physical assault (I twisted my partner’s arm or hair, I choked my partner, etc.), and sexual coercion (I insisted my partner have oral or anal sex but did not use physical force, I made my partner have sex without a condom, etc.) subscales. It also contains a seventh answer item, which represents ―Not in the past year, but it has happened before. Cronbach’s alpha for the present study was >0.77.

The Spanish adaptation of the Ambivalent Sexism Inventory (ASI) (Expósito et al. 1998) was employed for this study (Glick and Fiske 1996). This inventory consists of a 22-item self-report rated on a 6-point Likert-type scale (0 = strongly disagree; 5 = strongly agree). The test contains two scales: hostile (women are too easily offended, feminists are not seeking for women to have more power than men, etc.) and benevolent sexism (women, as compared to men, tend to have a more refined sense of culture and good taste, many women have a quality of purity that few men possess, etc.). A higher score is interpreted as having higher sexist attitudes. For this study we employed the total score by averaging both scales. Internal consistency for the total score in this study was 0.91.

### MRI data acquisition and analysis

Participants were scanned using Philips Achieva TX, 3 T using an 8-channel head coil with parallel acquisition technology (SENSE). All participants were instructed at the beginning of the acquisitions to avoid movements. The acquisition protocol consisted of a T1-weighted high-spatial resolution 3D gradient echo sequence with the following parameters: echo time = 3 s, repetition time = 6.2 s, flip angle = 100, voxel size = 1 × 1 × 1 mm^3^, and 6 min duration. The T2*-weighted MRI 2D EPI BOLD sequence was acquired with the following main parameters TE = 35 ms, TR = 2000 ms, temporal dynamics = 265, pixel size = 1.8 × 1.8 mm^2^, slice thickness = 5 mm, and a total duration of 9 min.

Cortical reconstruction and volumetric segmentation of the T1 images of the 117 patients were conducted using the FreeSurfer software package, specifically version 7.2.0. The processing flow employed for all subjects was “recon-all” with default parameters. The process began with image transformation to Talairach space, followed by intensity inhomogeneity correction, bias field correction (1), and skull separation (2). Subsequently, the white matter (WM) was separated from the gray matter (GM) and other tissues, and the volume was filled within the WM–GM boundary. The resulting surface was tessellated and smoothed. After these preprocessing steps, the surface was inflated and mapped onto a spherical atlas. This method adapted to the folding pattern of each individual brain, leveraging consistent folding patterns like the central sulcus and Sylvian fissure as landmarks, ensuring high localization accuracy. FreeSurfer employs a probabilistic approach based on Markov random fields for automatic labeling of brain regions using an atlas of 34 cortical regions. Cortical thickness was computed as the average distance between the WM–GM boundary and the pial surface on the tessellated surface.

#### Data analysis

After checking for normal distribution of data employing the Kolmogorov–Smirnov test (*P* ≤ .05), one-way ANOVAs and chi-squared tests were conducted to assess group differences in terms of age, sociodemographic variables, and sexism. The effect size for significant differences between groups was calculated with Cohen’s *d* (Cohen 1988) and Cramér’s V.

Regarding the first aim of the study, group differences in the cortical thickness of the frontal regions were calculated by applying one-way ANOVAs.

With regard to the second aim of this research, Pearson correlations between the cortical thickness of the frontal regions and sexism scores were calculated while controlling the effect of the control group. Afterwards, partial correlations were conducted including the role of group (dysexecutive versus non-dysexecutive versus controls) and IQ as covariates among previously significant models.

Statistical analyses were performed using the Statistical Package for the Social-Sciences 28.0 (SPSS IBM) software. All *P*-values, *P* ≤ 0.05 were considered significant.

## Results

As can be seen in Table 1, there were no “group” differences in sociodemographic variables, but groups differed in sexism scores (F = 16.73, *P* < 0.001, eta square = 0.227). Specifically, dysexecutive IPV perpetrators exhibited higher scores than IPV perpetrators without executive dysfunction (*t* = 7.11, *P* = 0.021) and controls (*t* = 11.69, *P* < 0.001).

**Table 1.** Means, standard deviations, percentages, and means comparisons for socio-demographic and psychological variables.

	IPV perpetrators dysexecutive (*n* = 38)	IPV perpetrators non-dysexecutive (*n* = 22)	Controls (*n* = 57)	F ANOVA/Chi-square	Significance	Effect size
Age (M, SD)	42.66 (11.38)	38.73 (11.39)	39.00 (14.39)	1.07	0.346	0.018
Nationality (%)						
Spanish	92	91	88	11.95	0.153	0.153
Latin Americans	5	5	9			
Other	3	4	3			
Level of education (%)						
Primary/lower secondary	63	45	42	8.47	0.090	0.190
Upper secondary	34	32	46			
University	3	23	12			
Laterality (%)						
Right-handed	92	91	88	0.516	0.772	0.066
Left-handed	8	9	12			
Sexism	28.16 (9.08)	21.05 (9.62)	16.46 (10.02)	16.73	0.000	0.227

Note. Abbreviations: M = Mean; SD = Standard deviation.

**Figure 1.**
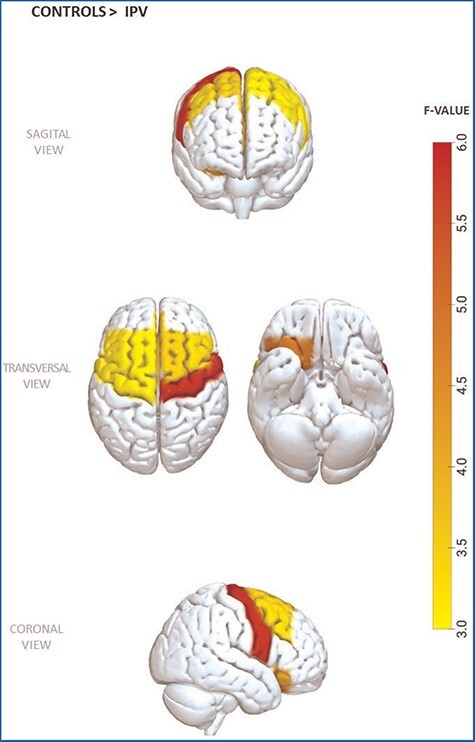
Cortical thickness differences in frontal regions between intimate partner violence perpetrators with executive dysfunctions in comparison with controls.

After checking for group differences in regional frontal cortical thickness, significant differences were found in the right rostral anterior cingulate (*F* = 3.74, *P* = 0.027, eta square = 0.062), right and left superior frontal (*F* = 3.67, *P* = 0.028, eta square = 0.061 and *F* = 3.15, *P* = 0.047, eta square = 0.052, respectively), right and left caudal middle frontal (*F* = 3.48, *P* = 0.034, eta square = 0.058 and *F* = 3.32, *P* = 0.040, eta square = 0.055, respectively), right medial orbitofrontal (*F* = 4.19, *P* = 0.018, eta square = 0.068), right paracentral (*F* = 3.64, *P* = 0.029, eta square = 0.060), and right and left precentral (*F* = 5.79, *P* = 0.004, eta square = 0.092 and *F* = 3.24, *P* = 0.043, eta square = 0.054, respectively). Post-hoc analysis revealed that IPV perpetrators with executive dysfunction show lower volume in the above-mentioned brain structures compared to controls (*P* < 0.05, for all cases), except for the right precentral region, where no significant differences between groups were found (*P* = 0.067) (Table 2 and Fig. 1).

**Table 2.** Cortical thickness differences in frontal areas between IPV perpetrators and controls classified according to their executive performance.

Region	IPV perpetrators dysexecutive (*n* = 38)	IPV perpetrators non-dysexecutive (*n* = 22)	Controls (*n* = 57)	F ANOVA	Significance	Effect size	Group differences
Rostral anterior cingulate (left)	2.65 (0.170)	2.67 (0.213)	2.69 (0.173)	0.744	0.478	0.013	
Rostral anterior cingulate (right)	2.65 (0.197)	2.69 (0.177)	2.76 (0.165)	3.74	**0.027**	0.062	Ac
Rostral middle frontal (left)	2.32 (0.131)	2.36 (0.105)	2.38 (0.097)	3.01	0.053	0.050	
Rostral middle frontal (right)	2.35 (0.126)	2.38 (0.110)	2.40 (0.103)	2.36	0.099	0.040	
Superior frontal (left)	2.66 (0.138)	2.69 (0.126)	2.73 (0.112)	3.67	**0.028**	0.061	ac
Superior frontal (right)	2.67 (0.136)	2.69 (0.119)	2.73 (0.116)	3.15	**0.047**	0.052	ac
Caudal anterior cingulate (left)	2.44 (0.217)	2.38 (0.175)	2.43 (0.181)	0.813	0.446	0.014	
Caudal anterior cingulate (right)	2.36 (0.165)	2.34 (0.184)	2.38 (0.154)	0.563	0.571	0.010	
Caudal middle frontal (left)	2.49 (0.134)	2.53 (0.103)	2.55 (0.137)	3.48	**0.034**	0.058	ac
Caudal middle frontal (right)	2.49 (0.128)	2.51 (0.137)	2.56 (0.109)	3.32	**0.040**	0.055	ac
Lateral orbitofrontal (left)	2.65 (0.121)	2.65 (0.128)	2.69 (0.141)	1.57	0.213	0.027	
Lateral orbitofrontal (right)	2.56 (0.154)	2.56 (0.133)	2.59 (0.161)	0.733	0.483	0.013	
Medial orbitofrontal (left)	2.39 (0.116)	2.38 (0.126)	2.44 (0.138)	2.34	0.101	0.039	
Medial orbitofrontal (right)	2.47 (0.127)	2.46 (0.138)	2.53 (0.114)	4.19	**0.018**	0.068	ac
Paracentral (left)	2.40 (0.153)	2.39 (0.151)	2.45 (0.144)	1.71	0.185	0.029	
Paracentral (right)	2.37 (0.144)	2.38 (0.148)	2.45 (0.144)	3.64	**0.029**	0.060	ac
Precentral (left)	2.49 (0.148)	2.51 (0.189)	2.59 (0.146)	5.79	**0.004**	0.092	ac
Precentral (right)	2.48 (0.139)	2.49 (0.173)	2.56 (0.157)	3.24	**0.043**	0.054	–

Note.Group differences: a (IPV perpetrators dysexecutive); b (IPV perpetrators non-dysexecutive); c (controls). The bold values are significant *P* values.

Regarding correlation analysis, some of the frontal brain structures exhibited a significant and inverse association with sexism scores in all the samples. Concretely, sexism was related to the rostral anterior cingulate (left) (*r* = −0.195, *P* = 0.035), rostral middle frontal (left) (*r* = −0.192, *P* = 0.038), rostral middle frontal (right) (*r* = −0.317, *P* = 0.001), superior frontal (left) (*r* = −0.256, *P* = 0.005), superior frontal (right) (*r* = −0.304, *P* = 0.001), caudal middle frontal (left) (*r* = −0.266, *P* = 0.004), caudal middle frontal (right) (*r* = −0.321, *P* < 0.001), medial orbitofrontal (right) (*r* = −0.308, *P* = 0.001), paracentral (left) (*r* = −0.191, *P* = 0.040), paracentral (right) (*r* = −0.312, *P* = 0.001), precentral (left) (*r* = −0.332, *P* < 0.001), and precentral (right) (*r* = −0.257, *P* = 0.005). When controlling the effect of group and IQ, only the following remained significant: rostral middle frontal (right) (*r* = −0.275, *P* = 0.003), superior frontal (right) (*r* = −0.244, *P* = 0.009), caudal middle frontal (left) (*r* = −0.224, *P* = 0.017), caudal middle frontal (right) (*r* = −0.268, *P* = 0.004), medial orbitofrontal (right) (*r* = −0.222, *P* = 0.018), paracentral (right) (*r* = −0.255, *P* = 0.006), precentral (left) (*r* = −0.240, *P* = 0.011), and precentral (right) (*r* = −0.198, *P* = 0.036).

## Discussion

Our results indicate that IPV perpetrators with executive dysfunction exhibit a smaller cortical thickness in multiple frontal regions and higher sexism levels compared to controls. IPV perpetrators without executive malfunctioning and controls exhibited similar scores. Most importantly, specific structure variations among the frontal and prefrontal regions that allowed to differentiate groups entailed higher sexism scores, such as thinner superior frontal (right), caudal middle frontal (right), medial orbitofrontal (right), paracentral (right) and precentral (left).

Regarding the first objective of this study, our results show that IPV perpetrators, in particular those exhibiting executive alterations, display a reduced cortical thickness in some frontal regions compared to non-violent men (control group). Furthermore, dysexecutive IPV perpetrators also had the highest levels of sexism. Therefore, our results use MRI techniques to confirm the presence of executive malfunctioning in IPV perpetrators, differences which are not a result of IPV perpetrators’ malingering responses (e.g. lying or exaggerating responses in neuropsychological tests). That is, these findings support the important role of executive dysfunctions, at least in part, in IPV perpetration (Horne et al. 2020, Humenik et al. 2020). In fact, it could be hypothesized that the maintenance or proneness to this type of violence might be explained by high sexism levels, as previously pointed out (Vitoria-Estruch et al. 2017, 2018). However, not all IPV perpetrators differed from controls, given how IPV perpetrators without executive alterations exhibited similar scores in sexism and cortical thickness.

In terms of all the frontal regions that allow distinguishing dysexecutive IPV perpetrators from controls, a previous study has signalled that IPV perpetrators tend to present a lower cortical thickness in the orbitofrontal, as well as in the anterior and posterior cingulate cortex compared to other types of criminals (Verdejo-Román et al. 2019). These results partly correspond with our data as IPV perpetrators with executive dysfunctions exhibited a thinner right rostral anterior cingulate and right medial orbitofrontal compared to controls. While the rostral anterior cingulate cortex is responsible for integrating emotion and cognition, as well as amygdala-dependent learning (Bissière et al. 2008), the right medial orbitofrontal cortex tends to play a significant role in associations related to rewarding processes (Rolls 2004). Therefore, both structures are crucial when trying to anticipate the consequences of behavior. However, these were not the only structures in this study which allowed to differentiate groups of IPV perpetrators with executive malfunctioning. These individuals also presented a thinner right and left superior frontal, right and left caudal middle frontal, right paracentral, right and left precentral, which is especially relevant for working memory, reorienting attention, and motor and sensory innervations, among other functions (Boisgueheneuc et al. 2006, Japee et al. 2015, Banker and Tadi 2022). Their role in IPV maintenance could be explored through the second objective of our study.

Concretely, it could be concluded that IPV perpetrators with executive dysfunctions exhibit the highest levels of sexism, which corresponds with previous conclusions in this field (Romero-Martínez et al. 2013, Vitoria-Estruch et al. 2017, 2018). In any case, the main novelty of the current study was to establish a link between sexism and the cortical thickness of frontal regions, while controlling the effect of group. Curiously, many of the above-described frontal brain regions were inversely related to sexism. That is, the thinner the cortical thickness, the higher the levels of sexism across groups. Therefore, the maintenance of certain rigid cognitive schemas (e.g. sexism) might be explained, at least in part, by executive malfunctioning. Nonetheless, it cannot be concluded that finding a brain correlate of these cognitive schemas means that they cannot be changed. Several empirical studies have pointed out that IPV perpetrators with good treatment adherence, who complete intervention programs, exhibit a decrease on their scores in sexism and attitudes towards IPV (Lila et al. 2013, 2020, Park and Kim 2023) and improve their executive functioning (Romero-Martínez et al. 2022a). Therefore, it would be necessary to apply MRI techniques to confirm whether those changes in sexism scores correspond to changes in frontal cortical thickness. This would help compare conclusions with therapists to assess the risk of recidivism after the intervention has ended. It would also avoid the same biases that can arise from social desirability or malingering responses in neuropsychological tests.

Despite the interest of the conclusions of this study, it is necessary to pay attention to several limitations that affect the external validity of these conclusions, and which could guide future research in this field. The first limitation refers to the sample size. Although this study employed more than 100 participants, it would be necessary to include a larger and more diverse range of participants to increase the external validity of our results. Moreover, we included a self-report and neuropsychological test with limitations such as social desirability and malingering responses. Hence, it would be necessary to cross-reference those results by using other instruments. Lastly, the limitations that affect the sensitivity of MRI techniques to assess cortical thickness should be considered. This should be taken into account in future empirical research and when implementing new projects to overcome current limitations.

Our research reinforces the importance of combining biological markers, such as neuroimaging techniques, with psychological instruments to accurately develop risk profiles of IPV perpetrators. Our study highlights the existence of brain differences in cortical thickness (e.g. frontal and prefrontal cortical thickness) that could be related to cognitive alterations in IPV perpetrators. However, these do not necessarily allow to differentiate all types of IPV perpetrators. Concretely, they allowed to distinguish those classified with special risk. Hence, it is necessary to be cautious and to complement these conclusions with different instruments, including the assessment of therapists. MRI techniques should be considered a complementary measurement to those conclusions provided by self-reports and neuropsychological assessments in order to better understand IPV perpetrator profiles. Therefore, future steps should be considered in combination with the variables mentioned above, to establish IPV subtypes for a good prediction of these key variables. In this regard, future empirical research should consider all these issues to reinforce neuroimaging results.

In summary, our research highlights the need for clinical psychologists, especially in the field of IPV, to combine their efforts with other professionals, such as psychobiologists, medical doctors, radiologists, among others, to adopt a multidisciplinary approach that allows us to better understand the complex phenomenon of IPV. The employment of neuroimaging techniques will help overcome specific tactics employed by IPV perpetrators, such as malingering their responses to certain self-reports or interviews. In this sense, our data offer a new perspective to give visibility to how their brains process surrounding information. Therefore, we think it would be necessary to incorporate these techniques before starting intervention programs to complement IPV profiles or subtypes, which in turn, might allow to clarify the therapeutic needs of these men and reinforce their adherence to interventions. Moreover, the current data strengthens specific brain circuitry which is relevant in terms of characterizing IPV perpetrators. Thus, this might guide the development of alternative treatment modules, which directly impact variations in cortical thickness, reducing in turn IPV perpetrators’ risk of recidivism.
