# Correction to: Executive dysfunction and cortical variations among intimate partner violence perpetrators and the association with sexism

**DOI:** 10.1093/scan/nsaf073

**Published:** 2025-09-02

This is a correction to: Ángel Romero-Martínez, María Beser-Robles, Leonor Cerdá-Alberich, Fernando Aparici, Luis Martí-Bonmatí, Carolina Sarrate-Costa, Marisol Lila, Luis Moya-Albiol, Executive dysfunction and cortical variations among intimate partner violence perpetrators and the association with sexism, *Social Cognitive and Affective Neuroscience*, Volume 19, Issue 1, 2024, https://doi.org/10.1093/scan/nsae046

In the originally published online version of this manuscript, there was an error in the **Funding** section. The text under this heading should read: “This research was partially supported by grant number PID2022-142287OA-I00; funded by MCIN/AEI/10.13039/501100011033 and by ERDF, EU and The Prometeo Program for research groups of excellence of the Ministry of Innovation, Universities, Science and Digital Society of the Generalitat Valenciana, grant number CIPROM/2021/46.”

This emendation is been outlined only in this correction notice to preserve the version of record.

